# Supplementary material for: Mechanisms governing the pioneering and redistribution capabilities of the non-classical pioneer PU.1
Source: Nat Commun. 2020 Jan 21;11:402. doi: 10.1038/s41467-019-13960-2 (PMC6972792; doi:10.1038/s41467-019-13960-2)
Supplement: Supplementary file 7 — Source data [file 41467_2019_13960_MOESM7_ESM.zip › Source_Data/FigureS2/FigureS2B_MotifScanOutput/KmeansCluster10/homerResults/motif1.similar.html]

motif1

## Information for motif1

A
T
G
C
C
T
G
A
A
T
G
C
G
C
A
T
C
G
A
T
A
T
G
C
A
G
T
C
G
A
C
T
A
G
T
C
G
C
A
T
G
A
C
T
G
A
C
T
  
Reverse Opposite:  

C
T
G
A
C
T
G
A
C
G
T
A
T
C
A
G
C
T
G
A
C
T
A
G
A
T
C
G
C
G
T
A
C
G
T
A
T
A
C
G
G
A
C
T
T
A
C
G
  

|  |  |
| --- | --- |
| p-value: | 1e-1669 |
| log p-value: | -3.845e+03 |
| Information Content per bp: | 1.611 |
| Number of Target Sequences with motif | 2865.0 |
| Percentage of Target Sequences with motif | 77.18% |
| Number of Background Sequences with motif | 4898.0 |
| Percentage of Background Sequences with motif | 11.08% |
| Average Position of motif in Targets | 102.3 +/- 30.6bp |
| Average Position of motif in Background | 101.0 +/- 72.8bp |
| Strand Bias (log2 ratio + to - strand density) | 0.0 |
| Multiplicity (# of sites on avg that occur together) | 1.23 |
| Motif File: | file (matrix) reverse opposite |

### Similar de novo motifs found

|  |  |  |  |  |  |  |  |
| --- | --- | --- | --- | --- | --- | --- | --- |
| Rank | Match Score | Redundant Motif | P-value | log P-value | % of Targets | % of Background | Motif file |
| 1 | 0.991 | A T G C C T G A A T G C C G A T C G A T A G T C A G T C A G C T A G T C G C A T A G C T | 1e-1587 | -3654.732368 | 83.76% | 16.33% | motif file (matrix) |
| 2 | 0.996 | A T G C A T G C C T G A A T G C C G A T A C G T A G T C A G T C A G C T A G T C G A C T G A C T G A C T | 1e-1574 | -3625.883468 | 81.52% | 14.97% | motif file (matrix) |
| 3 | 0.975 | C T G A T C A G T C G A A C T G A C T G C G T A C G T A T A C G G C A T T C A G | 1e-1453 | -3346.892919 | 73.33% | 11.65% | motif file (matrix) |
| 4 | 0.965 | T C G A T C G A C T A G A C T G C G T A C G T A T A C G G A C T T A C G | 1e-1343 | -3093.106584 | 70.12% | 11.35% | motif file (matrix) |
| 5 | 0.983 | A G T C A T G C G C T A A T G C C G A T A C G T A G T C A G T C A G C T A G T C G A C T G A C T G A C T A G C T | 1e-1295 | -2982.974905 | 82.54% | 20.37% | motif file (matrix) |
| 6 | 0.934 | T C G A A C T G A C T G C G T A C G T A A C T G A C G T T A C G | 1e-1157 | -2664.560827 | 62.07% | 9.65% | motif file (matrix) |
| 7 | 0.917 | G A T C A G T C A T G C C G T A A G T C A C G T C G A T T G A C A G T C A G C T G A T C G C A T A G C T G C A T | 1e-1127 | -2595.146361 | 65.33% | 11.77% | motif file (matrix) |
| 8 | 0.929 | C T G A A C T G A C T G C G T A C G T A A C T G A C G T | 1e-1114 | -2567.237250 | 62.28% | 10.33% | motif file (matrix) |
| 9 | 0.939 | T C G A A T G C G C A T C A G T A T G C A G T C G A C T T A G C G A C T G A C T G C A T | 1e-984 | -2265.870338 | 77.94% | 23.10% | motif file (matrix) |
| 10 | 0.902 | T C G A C T G A C G T A T C A G T C G A A T C G A C T G C G T A C G T A A C T G A G T C T A G C T C G A C A T G | 1e-904 | -2082.734018 | 73.17% | 21.10% | motif file (matrix) |
| 11 | 0.888 | C T G A A T G C A C G T A C G T A G T C A G T C A T G C G A C T G C T A G A C T | 1e-825 | -1900.093035 | 58.76% | 13.02% | motif file (matrix) |
| 12 | 0.901 | C T G A C T G A C G T A T A C G T G C A T C A G T C A G C T G A C G T A T A C G | 1e-811 | -1867.701698 | 69.40% | 20.41% | motif file (matrix) |
| 13 | 0.833 | A T G C G T C A A T G C G C A T C G A T A T G C G A T C A T C G A G T C G C A T G A C T G A C T | 1e-746 | -1718.580633 | 45.12% | 7.28% | motif file (matrix) |
| 14 | 0.923 | T G C A C G A T A T C G C T G A C T A G T A C G G T C A C G T A T A C G C G A T C A T G T C A G | 1e-735 | -1693.544353 | 45.04% | 7.39% | motif file (matrix) |
| 15 | 0.877 | A T G C G C A T A C G T A G T C A G T C G A C T T A G C G A C T A G C T | 1e-732 | -1686.213966 | 67.86% | 21.18% | motif file (matrix) |
| 16 | 0.868 | A T G C T G C A A T G C C G A T A C G T A G T C A G T C | 1e-681 | -1568.629474 | 65.84% | 20.97% | motif file (matrix) |
| 17 | 0.792 | A C T G C A T G G T C A C G T A T G A C G C A T A C T G T G A C | 1e-626 | -1443.027941 | 58.57% | 17.15% | motif file (matrix) |
| 18 | 0.820 | G A C T C T G A T A C G C T G A A C T G C G T A C G T A A C T G A C G T A T C G T C G A C G A T C A T G | 1e-625 | -1441.021986 | 46.77% | 10.03% | motif file (matrix) |
| 19 | 0.875 | T G A C C A T G C T G A A C T G C A T G G T C A T C G A A T C G A G C T T A C G A T G C C A G T C T G A | 1e-623 | -1435.355139 | 38.31% | 6.02% | motif file (matrix) |
| 20 | 0.866 | A C G T A C G T A G T C A G T C A G C T A T G C G A C T | 1e-591 | -1361.689490 | 74.92% | 31.40% | motif file (matrix) |
| 21 | 0.867 | A T G C T G C A A T C G A T C G G T C A C G T A T G A C A G C T T A C G | 1e-574 | -1321.831124 | 76.59% | 33.62% | motif file (matrix) |
| 22 | 0.885 | G A C T A G C T T A G C A G T C A G C T A G T C G A C T G A C T | 1e-573 | -1320.216183 | 63.79% | 22.40% | motif file (matrix) |
| 23 | 0.885 | C T G A A G T C A C G T A C G T A G T C A G T C A G C T A C T G G A C T A T G C G T C A T A G C | 1e-329 | -759.631829 | 30.58% | 7.47% | motif file (matrix) |
| 24 | 0.831 | C G A T A T G C G C T A T C A G A T C G A C T G C T G A T G C A A C T G A G C T A T G C T C G A | 1e-245 | -565.921379 | 16.65% | 2.63% | motif file (matrix) |
| 25 | 0.797 | C T A G A T C G T C A G A T C G C T G A C G T A T A C G C G A T | 1e-225 | -519.563428 | 39.68% | 16.40% | motif file (matrix) |
| 26 | 0.756 | T C G A C T G A G C T A T C A G T A G C C T A G A T C G G T C A C G T A | 1e-216 | -497.467282 | 23.55% | 6.45% | motif file (matrix) |
| 27 | 0.762 | A C G T A G T C A G C T C G A T A G T C A G T C G A C T A T G C G A C T C A G T A T G C G A T C G A T C | 1e-204 | -470.459798 | 44.07% | 20.66% | motif file (matrix) |
| 28 | 0.793 | A G T C C G A T A C G T A G T C A T G C A G C T A C T G A G T C | 1e-156 | -360.704306 | 22.84% | 7.81% | motif file (matrix) |
| 29 | 0.836 | C T G A G A T C C A G T C G A T T A G C A T G C G C A T A G T C C G A T A C G T | 1e-144 | -332.797687 | 8.54% | 1.06% | motif file (matrix) |
| 30 | 0.811 | A C T G A G T C A C G T A C G T A G T C A G T C A C G T A G T C | 1e-120 | -276.940787 | 20.58% | 7.74% | motif file (matrix) |
| 31 | 0.777 | C T G A T A C G C T G A A C T G A C T G T C G A C G T A C T A G T A G C G C A T C T G A T G A C | 1e-87 | -201.797455 | 10.56% | 2.99% | motif file (matrix) |
| 32 | 0.761 | G T C A C G T A G C T A T C G A T G C A A T C G C T A G T G C A C G T A A T G C T G C A | 1e-83 | -193.004688 | 10.45% | 3.04% | motif file (matrix) |
| 33 | 0.811 | C G T A A G T C A C G T A C G T A G T C A G T C A G T C A G T C A C G T | 1e-77 | -178.970769 | 4.07% | 0.40% | motif file (matrix) |
| 34 | 0.810 | G T C A C T G A G T A C G T A C C G T A C T A G A T C G C G T A C G T A T C A G C G A T A T C G A T G C G A T C | 1e-70 | -161.310781 | 4.04% | 0.48% | motif file (matrix) |
| 35 | 0.785 | A G T C A G T C A C G T A C G T A G T C G T A C A C G T A G T C A C G T G A T C T A C G T G A C | 1e-62 | -143.223345 | 10.21% | 3.62% | motif file (matrix) |
| 36 | 0.752 | C T G A C T G A T C G A G T C A C G T A T C A G C T G A A C T G A T C G C G T A C T G A G T C A A G T C G T C A | 1e-45 | -105.517203 | 5.90% | 1.74% | motif file (matrix) |
